# Four vertex technique for correcting urethral prolapse: technique description and cohort study

**DOI:** 10.3389/fsurg.2023.1149729

**Published:** 2023-06-13

**Authors:** Andrea Noya-Mourullo, Manuel Herrero-Polo, Oscar Heredero-Zorzo, Francisco García-Gómez, Carmen Urrea-Serna, Magaly-Teresa Marquez-Sanchez, Javier Flores-Fraile, Barbara-Yolanda Padilla-Fernandez, María-Fernanda Lorenzo-Gómez

**Affiliations:** ^1^ Urology Department of the University Hospital of Salamanca, Salamanca, Spain; ^2^ Department of Surgery of the University of Salamanca, Salamanca, Spain; ^3^ Urology Area, Department of Surgery of the University of La Laguna, Tenerife, San Cristobal de La Laguna, Spain

**Keywords:** urethral prolapse, woman, postmenopause, vertex, technique

## Abstract

**Introduction/Objectives:**

This study aims to describe the procedure and effectiveness of the four-vertex technique for correcting urethral prolapse in women.

**Methods and Materials:**

includes a retrospective case series of 17 patients who underwent surgery for urethral prolapse. Two study groups were distinguished based on the presence or absence of pelvic heaviness symptoms. The variables were analyzed, including age, BMI, concomitant diseases, obstetric and gynecological history, time from diagnosis to surgery, and outcomes of treatment.

**Results:**

All patients were postmenopausal, with a mean age at the time of the intervention of 70.41 years, with no differences between groups. Mean BMI was 23.67 kg/m2, higher in the group with a sensation of vaginal heaviness (*p* = 0.027). Mean time elapsed between diagnosis and operation was 231.58 days, with no differences between groups. Mean childbirth count was 2.29. The most frequent causes for consultation were urethrorrhagia (33.33%) and a bulging sensation (33.33%). After the intervention, 14 patients (82.35%) were asymptomatic, two (11.76%) had dysuria, and one (5.88%) had urinary urgency. Ten patients had pre-surgical urinary incontinence, which was resolved in nine patients. 17.46% subsequently presented pelvic organ prolapse. In three women there was secondary impairment of sexual activity.

**Conclusion:**

The four-vertex technique was effective in resolving symptoms in most patients. However, some patients experienced dysuria, urinary urgency, and pelvic organ prolapse after surgery. Urinary incontinence improved in most patients, but a few required additional treatments with suburethral tape. The study also identified associations between variables and the presence of cystocele, consultation for a bulging sensation, and bleeding from urethral prolapse. Overall, this study sheds light on the challenges and outcomes of surgical treatment for urethral prolapse and provides valuable insights for future research in this area.

## Introduction

Urethral prolapse is a condition that primarily affects postmenopausal women. It occurs when the distal urethral mucosa prolapses through the external urethral meatus. The incidence of urethral prolapse is rare, but it is more common in black women than in white women (1).

The etiology of urethral prolapse may be divided into congenital causes: presence of redundant urethral mucosa secondary to deficiency of the collagen supporting the urethral mucosa, which leads to urethral hypermobility and acquired causes as weakened attachment between the longitudinal and circular smooth muscle fibres of the urethra (2), which together with episodes of increased pressure on the abdominal cavity may eventually cause prolapse (3).

The risk factors described included recurrent urinary infections, abdominal trauma, burns, malnutrition, sexual abuse, oestrogen deficits (such as postmenopause, oophorectomy, chemotherapy), asthma, injuries and hernias, upper respiratory infections and preterm delivery are also more frequent in people who suffer from urethral prolapse (2).

Diagnosis is clinical and based on physical examination. Upon examination a fleshy, oedematous, congestive lesion, i.e., a mass with a central orifice, is observed surrounding the urethral meatus (4). If there are no other anomalies in the genitourinary tract, imaging tests are not indicated (3).

The surgical management of urethral prolapse aims to preserve the urethral length and to prevent recurrence of the prolapse. The four-flap technique is a surgical approach that has been described for the treatment of urethral prolapse. It involves the creation of four vaginal flaps that are sutured over the prolapsed urethral mucosa to provide support and fixation (5).

Initial treatment of prolapse is conservative and involves the attempt to manually reduce the prolapse, with local anaesthesia if necessary (6), warm sitz or hip baths, and cream with oestrogens (3) or corticosteroids to reduce inflammation, along with antibiotics to prevent superinfection of the prolapsed tissue (7), if previous treatment fail, or in the case of an incarcerated prolapse (4) or large prolapse, surgery is indicated (3).

Several studies have reported the successful use of the four-flap technique in the treatment of urethral prolapse. One study evaluated the surgical outcome and patient satisfaction of the four-flap technique and found that it was effective in treating urethral prolapse with a high rate of patient satisfaction (8). Another study reported the long-term outcomes of the four-flap technique in children with urethral prolapse and found that it was a safe and effective procedure with low rates of recurrence (9). Additionally, the four-flap technique has been used for the management of recurrent urethral prolapse with good outcomes (10).

Other surgical options include placing a ligature on a urethral catheter, electrocoagulation of the tissue, or cryosurgery (3). Some teams treat urethral prolapse with surgery 24 h after diagnosis, viewing surgery as the simplest, fastest and most effective treatment (11).

Urethral prolapse can cause significant morbidity and has been associated with incontinence, dysuria, and recurrent urinary tract infections. Management of this condition often requires surgical intervention, and there are several surgical techniques available. However, there is limited evidence to guide the choice of surgical technique, and more research is needed in this area (12).

Following surgery, the prolapsed urethral fragment is referred to anatomical pathology, which usually finds angiomatous vascular proliferation accompanied by vascular thrombosis and signs of urethritis.

A systematic review of the literature found that the four-vertex technique is an effective and safe procedure for the management of urethral prolapse in children (13). Additionally, a study reported the successful use of the four-flap technique in a small cohort of women with urethral prolapse (14).

This study focuses in describe the procedure and effectiveness of the four-vertex technique for correction of urethral prolapse in women who consult the Urology service and the association with risk and protective factors.

## Aims

1. To describe the procedure and effectiveness of the four vertex technique for correction of urethral prolapse.
2. To identify the variables influencing the outcomes of corrective surgery for urethral prolapse.

## Methods

A retrospective case series, observational, single-centre study was performed from 10/17/2000 to 04/15/2021.

Inclusion criteria: Age over 18 years old, with or without delivery history, women with surgery for urethral prolapse in the Urology Service of the Salamanca University Hospital, informed consent for the use of their clinical data for research purposes.

Exclusion criteria: Age under 18 years old, without surgery for urethral prolapse in the Urology Service of the Salamanca University Hospital, not informed consent for the use of their clinical data for research purposes.

In total, were identified 17 patients who had undergone surgery for urethral prolapse, and 19 procedures.

Two study groups were distinguished:

• GNF (no feeling of pelvic heaviness; *n* = 11): women with no feeling pelvic heaviness caused by the prolapse.
• GF (feeling of pelvic heaviness; *n* = 6): women with a feeling of pelvic heaviness caused by the prolapse.

### Variables included in the analysis

Age at the time of prolapse correction; Body Mass Index (BMI); concomitant diseases and treatments, toxic substance use, surgical history, obstetric and gynaecological history, physical status measured on the anaesthetic risk scale of the *American Society of Anesthesiologists* (ASA) to measured the pelvic pain, reason for consultation, time elapsed from diagnosis to surgery; size of resected tissue; results of pathology report; SF-36 quality of life survey (15) and on a survey designed to measure the outcomes of treatment for prolapse [symptoms related to prolapse (voiding difficulties, urinary incontinence, bulging sensation, bleeding, dyspareunia, urinary urgency, frequent urination or pollakiuria: see Annex 1)]: responses were evaluated before treatment or initial consult, one month after treatment, and at the end of the follow-up period; vulnerability measured with /active life VES-13 scale (16), patients with VES-13 score <3, considered non-vulnerable and identified in the tables as “fully active”; and patients with VES-13 score > 3, vulnerable and expressed in the tables as “restricted” or with limitations.

Descriptive statistics, univariate analysis Fisher's exact test for qualitative variables, comparation between the study groups and Student's t-test for quantitative variables. Multivariate logistic regression analysis was used to determine whether these variables act as protective factors or risk factors for the dependent variables. An omnibus test was performed for each case to assess the predictive capability of the regression model, which was found to be suitable for this analysis. All analyses were performed with IBM Corp. Released 2017. IBM SPSS Statistics for Windows, Version 25.0. Armonk, NY: IBM Corp, *p* < 0.05 was considered significant.

### Procedure

#### Preoperative preparation (preceded by preanaesthetic assessment)

**Figure 1:**
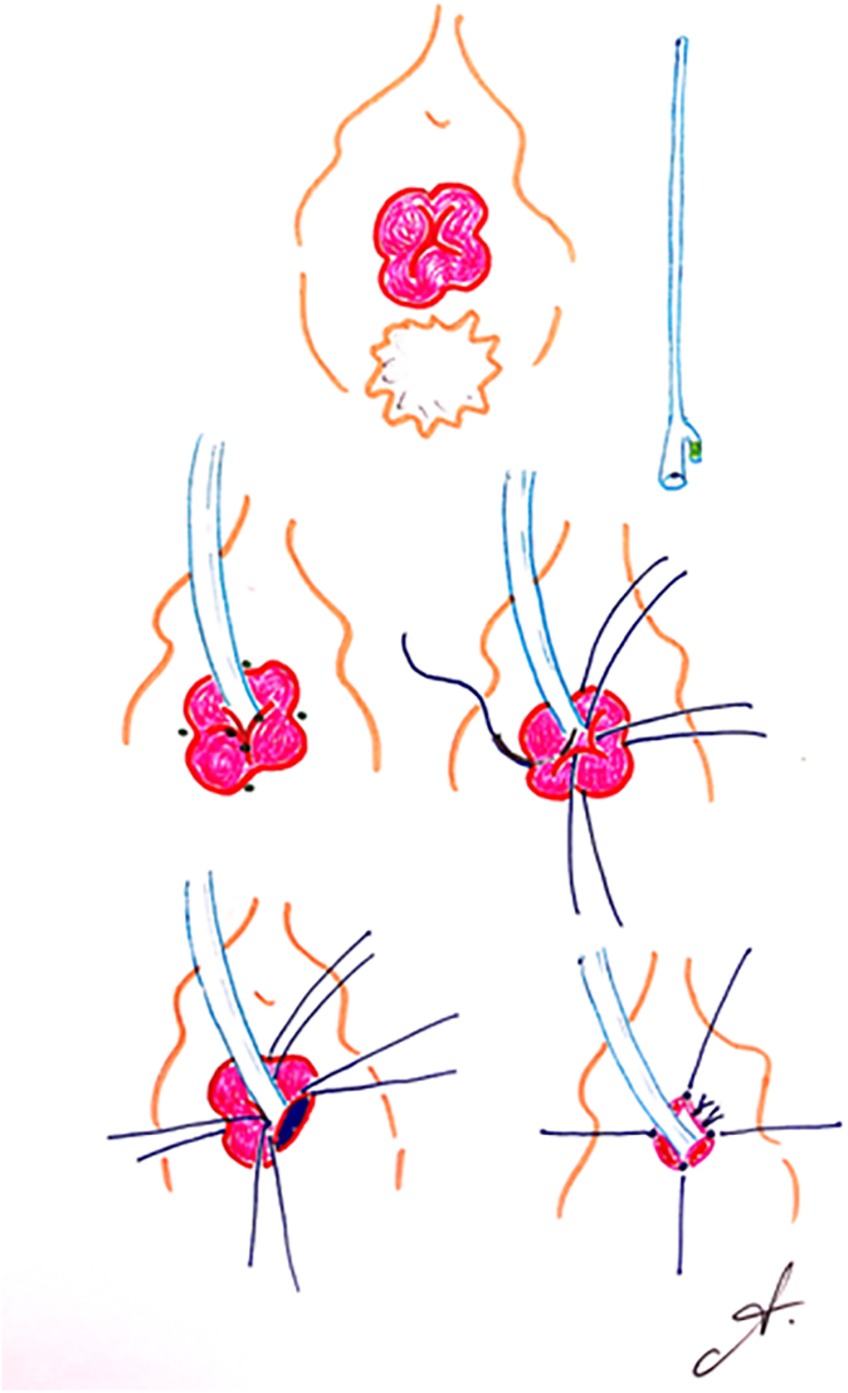
Four-point surgical technique. Figures by Dr Andrea Noya-Mourullo.

(Figure 1) The genital area must be shaved. Antibiotic prophylaxis is administered by a single dose of 2 grams of amoxicillin, and gastric protection is given by 40 mg of intravenous pantoprazole.

The procedure is generally performed under neuraxial anaesthesia, in the lithotomy position. Once the target area is disinfected with local antiseptic, a 16 Ch urethral catheter with a 20 cc balloon is fitted. The proximal urethral mucosa is gently avulsed. Presence of ureteral orifices is ruled out by meticulous inspection.

Multifilament absorbable stay sutures are placed at the 3, 6, 9 and 12 o'clock cystoscopic positions for the extirpation of the prolapse. The mucosa of each quadrant is extirpated using an electrosurgical unit with spatula electrode single-use handle. The healthy urethral mucosa is joined to the vaginal rim with discontinuous 4/0 absorbable sutures.

10 cc is withdrawn from the urinary catheter balloon and triantibiotic ointment is applied to the wound. Dressing and support garment are fitted.

Ethical considerations: The research project with code CEIm PI 2021 05 769 was approved on 24/05/2021 by the Ethics Committee for Clinical Research of the Salamanca Healthcare Area.

Costs: The study was financed by the Urological and Renal Multidisciplinary Research Group (GRUMUR) of the Salamanca Biomedical Research Institute (IBSAL). 37007 Salamanca, Spain.

## Results

Table 1 provides a summary of the characteristics of the sample groups. Age (mean 70.82, SD ± 8.01) in group GNF was higher with no significant difference (*p* = 0.754), there was significant difference in BMI (mean 23.01, SD ± 2.29), which was higher in the GF group (*p* = 0.027). The most frequent occupation was housewife with 58.82% in the overall sample. Additional information can be found in Supplementary Table S1.

**Table 1:** Distribution and comparison of the studied variables in the groups GNF and GF.

Groups	GNF, *n* = 11	GF, *n* = 6	Total, *n* = 17	*p*-value
Age (prolapse surgical correction)	70.82 ± 8.01	69.67 ± 4.88	70.41 ± 6.92	0.7540
BMI	23.01 ± 2.29	24.88 ± 1.86	23.67 ± 2.28	0.0270
Time between diagnosis and surgical correction (days)	188.36 ± 240.32	310.83 ± 344.86	231.67 ± 277.31	0.4520
Number of deliveries	2.45 ± 0.93	2.00 ± 1.41	2.29 ± 1.10	0.5250
Resected tissue size (mm)	45.83 ± 20.35	74.50 ± 50.20	53.00 ± 28.84	0.053

Age, body mass index (BMI), days elapsed between diagnosis and surgical correction of the prolapse, number of deliveries, and millimetres of resected tissue.

The most common reasons for consultation were urethrorrhagia (33.33%) and a sensation of bulging (33.33%) in the GF group (*p* = >0.050). Among the participants, 11.76% had haematuria and lower urinary tract symptoms (LUTS), 29.41% urethrorrhagia. Among the patients, 76.47% had a history of previous pregnancies, resulting in a total of 13 successful full-term deliveries, euthocic delivery was more frequent in the GNF group (90.91%, *p* = 0.0006), hysterectomy 17.65% (*p* = 0.514), caesarean section 17.65% (*p* = 1.0000) (Supplementary Table S1).

**Figure 2:**
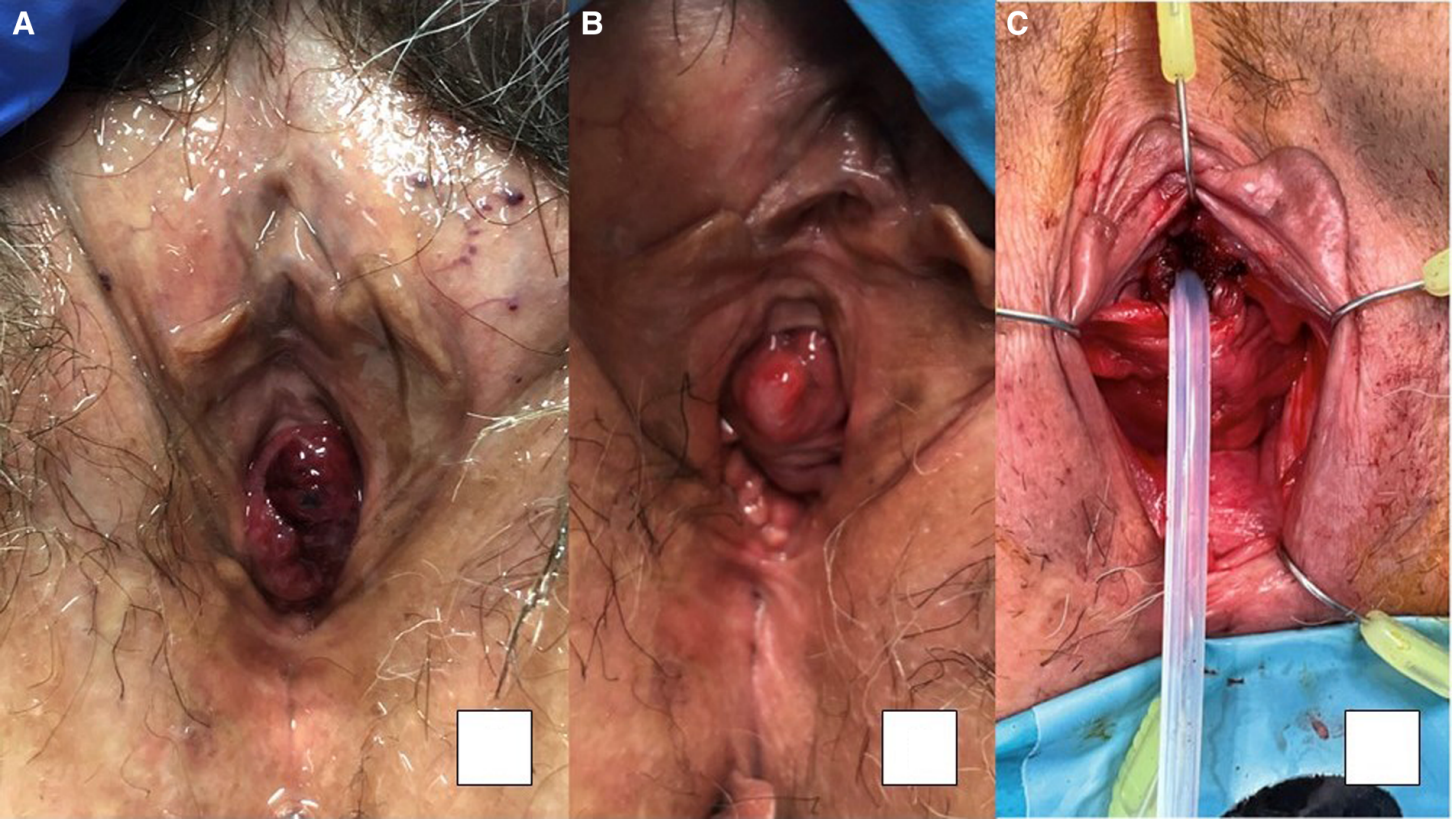
67-year-old multiparous woman. (**A**) thrombosed prolapse at diagnosis, (**B**) appearance at 21 days of pre-surgical follow-up; (**C**) results of surgical correction (Personal file of Dr Andrea Noya Mourullo).

The average time from prolapse diagnosis to surgical treatment was 231.67, SD ± 277.31 days, longer in the GF group with no significant difference (*p* = 0.452). Meatal stenosis was not observed in any patient. The cumulative incidence of diagnosed urethral prolapse in our healthcare area was 0.15% among the general population. Figure 2 illustrates the progression of urethral prolapse under conservative and surgical treatment. On average, 30.5 mm of urethral mucosa was resected, with a minimum of 10 mm in the GNF group and a maximum of 110 mm inn the GF group (mean 53 mm, SD ± 28.84, *p* = 0.053). Urothelial dysplasia was detected in only one case.

**Figure 3:**
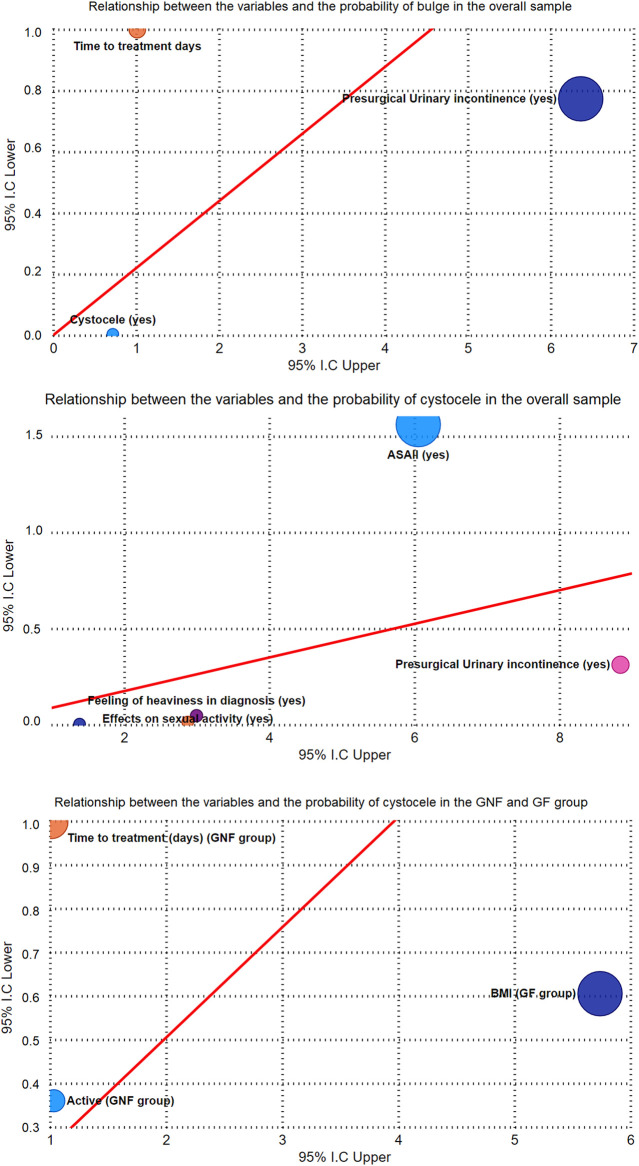
Relationship between the variables and the probability of presenting cystocele, bulging sensation as the reason for consultation, bleeding as the main symptom for consultation in urethral prolapse (multiple regression analysis).

After surgical treatment, symptoms resolved in 14 patients (82.35%), while 11.76% experienced dysuria, and 5.88% reported urinary urgency. Subsequently, 17.46% of patients developed pelvic organ prolapse with no significant difference between groups (*p* = >0.050).

Among the 10 patients (58.82%) who experienced urinary incontinence before surgery, only 11.76% had incontinence related to filling symptoms (*p* = > 0.050), which resolved on its own, and one patient (5.88%) had persistent incontinence that needed correction with suburethral tape (*p* = >0.050). Up to four patients (23.53%) temporarily relied on absorbent products, higher in the GNF group (27.27%). Additionally, three patients (17.65%) reported a decline in sexual quality of life following surgical prolapse correction, 33.33 more frequent in the GNF group (*p* = 0.514).

A logistic regression analysis was conducted to examine the relationship between studied variables and the presence of cystocele, consultation for a bulging sensation due to urethral prolapse, consultation for bleeding from urethral prolapse, and the number of deliveries in the patient’s obstetric history (Figure 3 and Table 2).

**Table 2:** Multivariate logistic regression analysis showing relative risk (RR) for the variables with a 95% confidence interval, and the following dependent variables: cystocele, bulge as reason for consultation, and haemorrhage as reason for consultation.

Relationship between the variables and the probability of cystocele in the overall sample						
Variables	B coefficient beta	Wald	*p*-value	RR:Relative risk	95% I.C Lower	95% I.C Upper
ASAII (yes)	3.466	5.057	0.025	32.000	1.561	6.054
Consultation for bulge (yes)	−2.773	3.075	0.008	0.062	0.003	1.386
Feeling of heaviness in diagnosis (yes)	−0.981	0.855	0.035	0.375	0.047	2.998
Effects on sexual activity (yes)	−1.609	1.400	0.023	0.200	0.014	2.876
Presurgical Urinary incontinence (yes)	1.099	0.905	0.034	3.000	0.312	8.841
Relationship between the variables and the probability of cystocele in the GNF group.						
Time to treatment (days)	0.005	1.847	0.174	1.005	0.998	1.012
Active	−0.981	2.099	0.0147	0.375	0.360	1.033
Relationship between the variables and the probability of cystocele in the GF group.						
BMI	0.622	1.175	0.027	1.863	0.605	5.738
Relationship between the variables and the probability of bulge in the overall sample.						
Cystocele (yes)	−2.996	4.851	0.028	0.050	0.003	0.719
Time to treatment days	0.006	3.174	0.0075	1.006	0.999	1.014
Presurgical Urinary incontinence (yes)	2.485	3.153	0.0076	12.000	0.773	6.362

The multivariate logistic regression analysis in the general sample revealed the following associations: patients with ASA II had a 32.00 times higher likelihood of showing cystocele (*p* = 0.025), while a decrease in pre-surgical urinary incontinence was associated with 3 times increased of cystocele (*p* = 0.034). When the reason for consultation was a bulging sensation, patients were 0.062 less likely to exhibit cystocele (*p* = 0.008), and if a feeling of heaviness was present at the time of diagnosis, the cystocele decreased by 0.375 times (*p* = 0.035). Additionally, an increase in the impact on sexual life resulted in a 0.200 decrease in the likelihood of cystocele (*p* = 0.023). Furthermore, as the time from diagnosis to surgical correction increased, patients were 1.006 more likely to report a bulging sensation (*p* = 0.007), and an increase in pre-surgical urinary incontinence led to a 12.00 higher likelihood of a bulge (*p* = 0.007). Conversely, the presence of cystocele reduced the likelihood of a bulge by 0.050 (*p* = 0.028). The presence of cystocele was associated with a 5.333-fold increased likelihood of bleeding (*p* = 0.012), and a decrease in the time from diagnosis to treatment resulted in a 0.989 lower likelihood of bleeding (*p* = 0.033). Finally, a decrease in pre-surgical urinary incontinence was associated with a 0.071 lower likelihood of bleeding (*p* = 0.005).

In GNF patients, the results of the multivariate analysis showed the following associations: an increase in the time from diagnosis to surgical treatment led to a 1.005 higher likelihood of cystocele (*p* = 0.004), while a decrease in physical activity was associated with a 0.375 lower likelihood of cystocele (*p* = 0.014). Similarly, an increase in the time from diagnosis to surgical treatment resulted in a 1.005 higher likelihood of a bulging sensation (*p* = 0.017), while a decrease in physical activity was associated with a 0.375 lower likelihood of a bulging sensation (*p* = 0.001). Furthermore, with an increase in age, patients were 1.609 more likely to exhibit bleeding (*p* = 0.007).

In GF, the multivariate analysis revealed the following findings: as BMI increased, patients had a 1.863 higher likelihood of presenting cystocele (*p* = 0.027), and as the number of deliveries increased, patients were 1.991 more likely to report a bulge (*p* = 0.032).

## Discussion

This study highlights the challenges associated with the diagnosis and treatment of urethral prolapse, a rare condition that is often underdiagnosed or misdiagnosed. Despite its simplicity in terms of diagnosis based on symptoms and examination, the condition remains poorly known. This lack of awareness contributes to delayed or inaccurate diagnoses, which can lead to complications and prolonged suffering for patients.

One interesting finding mentioned is that the number of pregnancies and vaginal deliveries does not appear to increase the risk of urethral prolapse. This goes against the common belief that childbirth is a significant contributing factor to the condition. Surprisingly, almost half of the cases occurred in nulliparous women, indicating that other factors may be at play in the development of urethral prolapse in comparison to a study by Ramirez-Garcia I, et al., the findings align with the results, emphasizing that nulliparity (having no previous pregnancies) does not increase the risk of urethral prolapse, these findings further support the notion that childbirth alone may not be a primary risk factor for the condition (17).

The treatment options for urethral prolapse, with manual reduction of the prolapse being an initial procedure to reduce swelling and minimize the risk of mucosal necrosis. Anesthetic lubricants and anti-inflammatory drugs are used to facilitate the reduction and alleviate pain. Surgical excision of the prolapsed tissue and reconstruction of the urethral mucosa are considered the definitive treatments, but they require careful consideration, particularly in cases of large prolapses that could displace the urethral meatus.

Complications associated with urethral prolapse include urinary incontinence and the potential for meatal stenosis during surgical anastomosis, the possible connection between urethral prolapse and pelvic organ prolapse, suggesting the importance of referring patients with urethral prolapse to pelvic floor rehabilitation units. This interdisciplinary approach can provide comprehensive care and address any associated pelvic floor dysfunction. In resemblance with the treatment, a study from Volloyhaug I et al., focuses on the surgical management of urethral prolapse. The study enhanced the importance of surgical intervention for cases of urethral prolapse that do not respond to conservative measures, it highlights the need for individualized treatment plans, considering factors such as the severity of the prolapse, associated pelvic floor disorders, and patient preferences. The review also provides an overview of various surgical techniques employed, such as vaginal or abdominal approaches, and emphasizes the importance of long-term follow-up to assess treatment outcomes (18).

The relationship between urethral prolapse, urinary incontinence, and concomitant cystocele. The incidence of cystocele is higher in women with higher ASA II classification and those experiencing urinary incontinence prior to surgery. This indicates a strong link between these conditions, emphasizing the need for a thorough evaluation and appropriate management of associated pelvic floor disorders, balancing with Anger JT, et al. confirms the strong link between these conditions, women a higher ASA II classification and those experiencing urinary incontinence prior to surgery have a higher incidence of cystocele. This finding underscores the importance of thoroughly evaluating and managing associated pelvic floor disorders, considering the interconnectedness of these conditions (19).

Furthermore, correlations between the presence of cystocele and various factors. For instance, in women without a feeling of heaviness, the probability of developing cystocele increases over time from diagnosis to corrective surgery. Lower levels of physical activity are associated with a decreased likelihood of experiencing a bulging sensation. Thomas AM, et al. find that lower levels of physical activity are associated with a decreased likelihood of experiencing a bulging sensation, indicating a potential relationship between lifestyle factors and the development or progression of cystocele. These findings underscore the importance of monitoring patients' symptoms and considering individual factors in the management of urethral prolapse (20).

Additionally, the impact of the number of deliveries on the presence of cystocele, revealing a negative correlation is observed, higher number of deliveries may reduce the risk of cystocele, this relationship differs among groups, in women with a feeling of heaviness, a positive correlation is observed between the age at diagnosis and the number of deliveries, indicating that older patients tend to have more deliveries. These findings highlight the complex interplay between age, childbirth, and the development of cystocele, necessitating further investigation. The Thomas AM, et al. study emphasizing the need for further investigation to better understand these relationships (20).

Lastly, the relationship between profession and the number of childbirths in women with urethral prolapse. The data suggest that shop assistants have undergone fewer deliveries compared to housewives, with a significant correlation between profession and the number of deliveries. This association raises interesting questions about potential socioeconomic factors that could influence the number of childbirths and subsequently impact the development of urethral prolapse. Further research is necessary to explore the underlying reasons behind this relationship and its implications (20).

This work contributes to the existing literature by providing information on the effectiveness of the four-vertex technique to correct urethral prolapse by identifying the variables that most influence the pre- and post-surgical results (gynecologic, obstetric, and medical history, age, hematuria, urethrorrhagia, bulging sensation). The combination of the specific technique, the analysis of the variables and the comparison with previous studies adds novelty to this research by proposing surgical treatment as a more effective treatment in the long term.

## Conclusions

This study provides valuable insights into urethral prolapse, including underdiagnosis, clinical presentation, treatment options, complications, and correlations with factors like age, childbirth, physical activity, and profession. The findings underscore the significance of increased awareness, early recognition, and comprehensive management for better patient outcomes and quality of life. Improves our understanding of urethral prolapse, its risk factors, and surgical management options. Additionally, highlights the correlation between cystocele and factors such as age, physical activity, and the number of deliveries, emphasizing the need for individualized care for the development, progression and optimize treatment strategies.

### Limitations and futher considerations

A larger sample size would provide more extensive results, include a control group to compare the outcomes and draw definitive conclusions about the associations between variables, provide a long-term follow-up to assess the durability of treatment outcomes or the potential for recurrence of symptoms or complications.

## Data Availability

The original contributions presented in the study are included in the article/**Supplementary Material**, further inquiries can be directed to the corresponding author/s.

## Supplementary material

SUPPLEMENTARY TABLE S1

Distribution and comparison of concomitant diseases, concomitant treatment, physical status, vulnerability, gynaecological and obstetric history in the groups GNF and GF.
