# Supplementary material for: Four vertex technique for correcting urethral prolapse: technique description and cohort study
Source: Front Surg. 2023 Jun 13;10:1149729. doi: 10.3389/fsurg.2023.1149729 (PMC10293759; doi:10.3389/fsurg.2023.1149729)
Supplement: Supplementary file 1 [file Table1.docx]

**Supplementary Table 1:** Distribution and comparison of concomitant diseases, concomitant treatment, physical status, vulnerability, gynaecological and obstetric history in the groups GNF and GF.

| Groups | GNF, n=11 | | GF, n=6 | | Total, n=17 | | p-value |
| --- | --- | --- | --- | --- | --- | --- | --- |
| Obstetric background | n | % | n | % | n | % | p-value |
| Euthocic delivery | 10 | 90.91 | 0 | 0.00 | 10 | 58.82 | 0.0006 |
| Dystocic delivery | 2 | 18.18 | 1 | 16.67 | 3 | 17.65 | 1.0000 |
| Hysterectomy | 1 | 9.09 | 2 | 33.33 | 3 | 17.65 | 0.5147 |
| Curettage | 2 | 18.18 | 1 | 16.67 | 3 | 17.65 | 1.0000 |
| ASA | n | % | n | % | n | % | p-value |
| ASA I | 3 | 27.27 | 0 | 0.00 | 3 | 17.65 | 0.5147 |
| ASA II | 6 | 54.55 | 3 | 50.00 | 9 | 52.94 | 1.0000 |
| ASA III | 2 | 18.18 | 3 | 50.00 | 5 | 29.41 | 0.2801 |
| Main symptom for consultation | n | % | n | % | n | % | p-value |
| Bulk | 3 | 27.27 | 2 | 33.33 | 5 | 29.41 | 1.0000 |
| Lower urinary tract symptoms | 2 | 18.18 | 0 | 0.00 | 2 | 11.76 | 0.5147 |
| Haematuria | 2 | 18.18 | 0 | 0.00 | 2 | 11.76 | 0.5147 |
| Urethrorrhagia | 3 | 27.27 | 2 | 33.33 | 5 | 29.41 | 1.0000 |
| Profession | n | % | n | % | n | % | p-value |
| Professor | 1 | 9.09 | 0 | 0.00 | 1 | 5.88 | 1.0000 |
| Cleaner | 3 | 27.27 | 0 | 0.00 | 3 | 17.65 | 0.5147 |
| Housewife | 6 | 54.55 | 4 | 66.67 | 10 | 58.82 | 1.0000 |
| Dressmaker | 1 | 9.09 | 0 | 0.00 | 1 | 5.88 | 1.0000 |
| Cystocele | 3 | 27.27 | 3 | 50.00 | 6 | 35.29 | 0.6000 |
| ECOG | n | % | n | % | n | % | p-value |
| Full active | 9 | 81.82 | 4 | 66.67 | 13 | 76.47 | 0.5840 |
| Restricted | 2 | 18.18 | 2 | 33.33 | 4 | 23.53 | 0.5840 |
| General health | n | % | n | % | n | % | p-value |
| Asynthomatic | 10 | 90.91 | 4 | 66.67 | 14 | 82.35 | 0.5147 |
| Independent | 7 | 63.64 | 2 | 33.33 | 9 | 52.94 | 0.3348 |
| Active | 3 | 27.27 | 0 | 0.00 | 3 | 17.65 | 0.5147 |
| Urinary incontinence | n | % | n | % | n | % | p-value |
| Urinary incontinence prior to prolapse surgery | 6 | 54.55 | 4 | 66.67 | 10 | 58.82 | 1.0000 |
| Urinary incontinence after prolapse surgery | 1 | 9.09 | 0 | 0.00 | 1 | 5.88 | 1.0000 |
| Absorbent use | 3 | 27.27 | 1 | 16.67 | 4 | 23.53 | 1.0000 |
| Impaired sexuality after prolapse surgery | 1 | 9.09 | 2 | 33.33 | 3 | 17.65 | 0.5147 |

UTI: Urinary tract infection, HT: hypertension, DM2: Diabetes Mellitus, ENT:Ear, nose and throat specialist, AMI: acute myocardial infarction. ACEI: angiotensin-converting enzyme inhibitor, AIIRA: Angiotensin II receptor antagonist. TOT: transobturator tape.
