# Supplementary material for: Four vertex technique for correcting urethral prolapse: technique description and cohort study
Source: Front Surg. 2023 Jun 13;10:1149729. doi: 10.3389/fsurg.2023.1149729 (PMC10293759; doi:10.3389/fsurg.2023.1149729)
Supplement: Supplementary file 2 [file Table2.docx]

**Supplementary table 2**. Identification of risk factors and protective factors with respect to the dependent variables cystocele, bulge as reason for consultation, and haemorrhage as reason for consultation.

| Risk factor | RR | | | p-value | | | | Protective factor | | | | RR | | | | p-value |
| --- | --- | --- | --- | --- | --- | --- | --- | --- | --- | --- | --- | --- | --- | --- | --- | --- |
| Cystocele: risk factors and protective factors in the overall sample | | | | | | | | | | | | | | | | |
| ASA II | 32.000 | | | 0.025 | | | | Bulge consultation (yes) | | | | 0.062 | | | | 0.008 |
| Presurgical urinary incontinence | 3.000 | | | 0.034 | | | | Feeling weight in diagnosis (yes) | | | | 0.375 | | | | 0.035 |
|  |  | | |  | | | | Affection sexual sphere (yes) | | | | 0.200 | | | | 0.023 |
| Cystocele: risk factors and protective factors in GNF | | | | | | | | | | | | | | | | |
| Time to treatment | 1.005 | | | 0.004 | | | | Active | | | | 0.375 | | | | 0.014 |
| Cystocele: risk factors and protective factors in GF | | | | | | | | | | | | | | | | |
| BMI | 1.863 | | | 0.027 | | | | none | | | | -- | | | | -- |
| Bulge as reason for consultation: risk factors and protective factors in the overall sample | | | | | | | | | | | | | | | | |
| Time to treatment | | 1.006 | | | 0.0075 | | Cystocele | | 0.050 | | | | 0.028 | | | |
| Presurgical urinary incontinence | | 12.000 | | | 00.76 | |  | |  | | | |  | | | |
| Bulge as reason for consultation: risk factors and protective factors in GNF | | | | | | | | | | | | | | | | |
| Time to treatment | | 1.005 | 0.017 | | | Active | | | | | 0.375 | | | | 0.001 | |
| Bulge as reason for consultation: risk factors and protective factors in GF | | | | | | | | | | | | | | | | |
| Number of deliveries | | 1.991 | 0.032 | | | None | | | | | -- | | | | -- | |
| Bulge as reason for consultation: risk factors and protective factors in GF | | | | | | | | | | | | | | | | |
| Cystocele | | 5.333 | 0.012 | | | Time to treatment (days) | | | | 0.989 | | | | 0.033 | | |
| Presurgical urinary incontinence (yes) | | 0.071 | 0.005 | | |  | | | |  | | | |  | | |
| Haemorrhage as reason for consultation: risk factors and protective factors in GNF | | | | | | | | | | | | | | | | |
| Age today | | 1.609 | 0.007 | | | Time to treatment in days | | | | | 0.988 | | | | 0.005 | |
|  | |  |  | | | Presurgical Urinary incontinence (yes) | | | | | 0.062 | | | | 0.008 | |

RR: relative risk. ASA: American Society of Anesthesiologists.
