# Supplementary material for: Four vertex technique for correcting urethral prolapse: technique description and cohort study
Source: Front Surg. 2023 Jun 13;10:1149729. doi: 10.3389/fsurg.2023.1149729 (PMC10293759; doi:10.3389/fsurg.2023.1149729)
Supplement: Supplementary file 3 [file Table3.docx]

**Supplementary Table 3.** Multivariate regression analysis of direct or inverse (positive or negative) correlation between variables and number of deliveries.

| **Variables** | **Unstandardized B Coefficients** | **Standardized B Coefficients** | | **p-value** | **95,0% Confidence Interval: Lower Bound** | **95,0% Confidence Interval: Upper Bound** |
| --- | --- | --- | --- | --- | --- | --- |
| Correlation between variables and number of deliveries in the overall sample | | | | | | |
| ASA III | 3.667 | 0.124 | | 0.021 | 0.637 | 6.697 |
| Cystocele cod | -0.455 | -0.203 | | 0.003 | -1.663 | 0.754 |
| Feeling of heaviness during diagnosis cod | 2.909 | -0.038 | 0.003 | | 1.175 | 4.643 |

| Correlation between variables and number of deliveries in GNF | | | | | |
| --- | --- | --- | --- | --- | --- |
| Age at diagnosis | 0.152 | 1.305 | 0.032 | 0.017 | 0.288 |
| Age today | -0.136 | -1.105 | 0.0059 | -0.278 | 0.007 |
| Effects on sexual activity | 1.700 | 0.549 | 0.0080 | -0.253 | 3.653 |
| Correlation between variables and number of deliveries in GF | | | | | |
| Shop assistant | -2.250 | -0.822 | 0.045 | -4.417 | -0.083 |
| Housewife | 1.750 | 0.600 | 0.020 | -1.487 | 4.987 |
