# Supplementary material for: Four vertex technique for correcting urethral prolapse: technique description and cohort study
Source: Front Surg. 2023 Jun 13;10:1149729. doi: 10.3389/fsurg.2023.1149729 (PMC10293759; doi:10.3389/fsurg.2023.1149729)
Supplement: Supplementary file 4 [file Table4.docx]

Appendix 1.- Survey on specific signs and symptoms of urethral prolapse:

**First visit – initial visit**

**Regarding your urethral prolapse, please answer the following questions, where 0 is “never” and 5 is “very frequently”.**

Do you experience voiding difficulties as a consequence of urethral prolapse?

Do you experience urinary incontinence as a consequence of or related to urethral prolapse?

Do you feel a sensation of a perineal lump resulting from, or related to, urethral prolapse?

Do you experience urethral bleeding or urethrorrhagia as a consequence of or related to urethral prolapse?

Do you experience dyspareunia resulting from, or related to, urethral prolapse?

Do you have urinary urgency resulting from, or related to, urethral prolapse?

Do you experience frequent urination resulting from, or related to, urethral prolapse?

**Follow-up visit one month after surgery:**

**Regarding your urethral prolapse, please answer the following questions, where 0 is “never” and 5 is “frequently”.**

Do you experience voiding difficulties as a consequence of urethral prolapse?

Do you experience urinary incontinence as a consequence of or related to urethral prolapse?

Do you feel a sensation of a perineal lump resulting from, or related to, urethral prolapse?

Do you experience urethral bleeding or urethrorrhagia as a consequence of or related to urethral prolapse?

Do you experience dyspareunia resulting from, or related to, urethral prolapse?

Do you have urinary urgency resulting from, or related to, urethral prolapse?

Do you experience frequent urination resulting from, or related to, urethral prolapse?

**Follow-up visit six months after surgery:**

**Regarding your urethral prolapse, please answer the following questions, where 0 is “never” and 5 is “frequently”.**

Do you experience voiding difficulties as a consequence of urethral prolapse?

Do you experience urinary incontinence as a consequence of or related to urethral prolapse?

Do you feel a sensation of a perineal lump resulting from, or related to, urethral prolapse?

Do you experience urethral bleeding or urethrorrhagia as a consequence of or related to urethral prolapse?

Do you experience dyspareunia resulting from, or related to, urethral prolapse?

Do you have urinary urgency resulting from, or related to, urethral prolapse?

Do you experience frequent urination resulting from, or related to, urethral prolapse?

**Final follow-up visit:**

**Regarding your urethral prolapse, please answer the following questions, where 0 is “no” and 5 is “frequently”.**

Do you experience voiding difficulties as a consequence of urethral prolapse?

Do you experience urinary incontinence as a consequence of or related to urethral prolapse?

Do you feel a sensation of a perineal lump resulting from, or related to, urethral prolapse?

Do you experience urethral bleeding or urethrorrhagia as a consequence of or related to urethral prolapse?

Do you experience dyspareunia resulting from, or related to, urethral prolapse?

Do you have urinary urgency resulting from, or related to, urethral prolapse?

Do you experience frequent urination resulting from, or related to, urethral prolapse?
